# Prognostic value of early bone marrow MRD status in CAR-T therapy for myeloma

**DOI:** 10.1038/s41408-023-00820-y

**Published:** 2023-04-05

**Authors:** Radhika Bansal, Mizba Baksh, Jeremy T. Larsen, Matthew A. Hathcock, David Dingli, A. Keith Stewart, Prashant Kapoor, Taxiarchis Kourelis, Suzanne R. Hayman, Rahma M. Warsame, Rafael Fonseca, P. Leif Bergsagel, Sikander Ailawadhi, Shaji K. Kumar, Yi Lin

**Affiliations:** 1 Division of Hematology, Mayo Clinic, Rochester, MN USA; 2 Division of Hematology and Oncology, Mayo Clinic, Jacksonville, FL USA; 3 Division of Hematology and Oncology, Mayo Clinic, Phoenix, AZ USA; 4 Princess Margaret Cancer Center, Toronto, ON Canada; 5 Division of Hematology and Oncology, Mayo Clinic, Scottsdale, AZ USA

**Keywords:** Myeloma, Disease-free survival

## Abstract

Bone marrow (BM) assessment of minimal residual disease (MRD) is prognostic for survival in multiple myeloma (MM). BM is still hypocellular at month 1 post CAR-T, thus the value of MRD negative (MRDneg) status at this timepoint is unclear. We examined the impact of month 1 BM MRD status in MM patients who received CART at Mayo Clinic between 8/2016 and 6/2021. Among 60 patients, 78% were BM-MRDneg at month 1; and 85% (40/47) of these patients also had decreased to less than normal level of both involved and uninvolved free light chain (FLC < NL). Patients who achieved CR/sCR had higher rates of month 1 BM-MRDneg and FLC < NL. The rate of sustained BM-MRDneg was 40% (19/47). Rate of conversion from MRDpos to MRDneg was 5%(1/20). At month 1, 38%(18/47) of the BM-MRDneg were hypocellular. Recovery to normal cellularity was observed in 50%(7/14) with a median time to normalization at 12 months (range: 3–Not reached). Compared to Month 1 BM-MRDpos patients, patients who were BM-MRDneg had longer PFS irrespective of BM cellularity [PFS: 2.9 months (95% CI, 1.2-NR) vs. 17.5 months (95% CI, 10.4-NR), *p* < 0.0001]. Month 1 BM-MRDneg and FLC below normal were associated with prolonged survival. Our data support the continued evaluation of BM early post-CART infusion as a prognostic tool.

## Introduction

Advances in diagnostic technologies have allowed defining minimal residual disease (MRD) disease status in patients with multiple myeloma (MM). The international myeloma working group (IMWG) had published guidelines for MRD assessment to identify depth of treatment response and allow uniform reporting within and outside clinical trials [1]. Serial, sustained BM-MRDneg through 12 months has been prognostic for prolonged progression-free survival across a variety of myeloma therapy [2].

Chimeric antigen receptor T (CAR-T) cell therapy has emerged as a potent treatment strategy against B-cell neoplasms with impressive outcomes and manageable toxicity [3–7]. In patients with MM receiving CAR-T on clinical trials, BM MRD assessment has been done on a scheduled basis regardless of M protein levels and IMWG response. BM clearance of plasma cells can be detected by month 1 post CAR-T, even before monoclonal protein disappears. This may be due to the mechanism of direct killing of plasma cells by CAR-T and the relatively slower clearance of M protein. It is important to note that BM may still be hypocellular at month 1 post-CAR-T, thus the prognostic value of BM MRD status at this early time point in therapy and when the detection level is limited is unclear. We examined the association of BM-MRD at month 1 with response, progression-free survival (PFS), and overall survival (OS) for patients who received CAR-T at Mayo Clinic.

## Methods

We conducted a retrospective review of electronic medical records of patients who received CAR-T for treatment MM between 8/2016 and 1/2021 at Mayo Clinic, Rochester, Arizona, and Florida. We selected all consecutive patients treated during the specific timeframe and excluded those treated in phase I clinical trials. CAR-T outcomes were obtained from the prospectively collected and validated immune effector cell program clinical database after IRB approval from Mayo Clinic. Informed consent was obtained from all subjects. Free light chains (FLC) at month 1 were defined as below normal or normal/elevated.BM cellularity and MRD assessment by flow cytometry were collected from clinical pathology reports at months 1, 3, 6, and 12. The sensitivity of this assay is 10–5 if adequate cell number has been acquired (minimum 2 × 10^6^ events) [8]. BM cellularity was defined as hypocellular or normocellular/ Not hypocellular as outlined in clinical pathology BM reports. Sustained BM Hypocellularity was defined as maintenance of BM hypocellularity confirmed ≥12 months apart with no normocellular/ hypercellular BM in between.

Best response were assessed as per IMWG criteria. Progression-free survival (PFS) was defined from CAR-T infusion to progressive disease or next treatment or death from any cause, whichever occurred first. Overall survival (OS) was measured from the date of CAR-T infusion to the date of death or last follow-up. PFS and OS were analyzed using Kaplan-Meier analysis and statistical significance were assessed using the two-tailed log-rank test. *χ*^2^-test was used to determine relationships between categorical variables. The Kruskal–Wallis rank sum test was used to determine associations between continuous variable and categories, and Pearson’s chi-square test was used to evaluate associations for categorical variables.

## Results

### Patient characteristics and clinical outcome

Among the 62 patients who received CAR-T for MM, 60 MM patients had adequate BM for clinical assessment at month 1. The median age at the time of CAR-T evaluation for this cohort was 62 (range, 34–81) years and 78% were males. All patients received lymphodepletion chemotherapy with fludarabine and cyclophosphamide from day −5 to day −3. The incidence of CRS was 75%, 5% for grade 3 or higher. The incidence for ICANS was 22%, and 2% for grade 3 or higher (Table 1). The CR/sCR rate was 45%. With a median follow-up of 20 months (range: 6.9–58.9 months), the median PFS and OS were 10.4 (95% CI: 6–17.9) months and 28.3 (95% CI: 21.9-not reached) months, respectively.

**Table 1:** Patient demographics and clinical outcomes.

Demographics	All patients (*N* = 60)	BM-MRDneg (*N* = 47)	BM-MRDpos (*N* = 13)	*P*- Value
Age (year), median (range)	62 (34–81)	62 (41–81)	60 (34–70)	0.3
Male, *n* (%)	32 (53)	23 (49)	9 (69)	0.2
Median time from initial diagnosis to CAR-T evaluation, year, median (range)	6 (0–15)	6 (0–15)	6 (0–15)	0.7
High risk cytogenetic profile, n(%)	28 (70)*	21 (70)	7 (70)	1
ISS Stage II/III, *n* (%)	14 (31)	11 (31)	3 (30)	0.9
ECOG ≥ 1, *n* (%)	17 (31)**	12 (29)	5 (39)	0.5
Bridging therapy, *n* (%)	46 (77)	39 (83)	7 (54)	**0.03**
Prior lines of therapy, median (range)	5 (2–17)	5 (2–12)	6 (2–17)	0.09
Plasmacytoma, *n*(%)	23 (38)	17 (36)	6 (46)	0.7
Clinical outcome				
Cytokine release syndrome, all grade, *n* (%)	45 (75)	36 (77)	9 (69)	0.6
ICANS, all grade, *n*(%)	13 (22)	11 (23)	2 (15)	0.5
Tocilizumab use, *n*(%)	25 (42)	22 (47)	3 (23)	0.1
Steroid use, *n*(%)	15 (25)	13 (28)	2 (15)	0.4
Cumulative dexamethasone dose, mg, median (range)	20 (10–1309.6)	20 (10–1309.6)	25 (10–40)	1
Anakinra use, *n*(%)	5 (8)	5 (10.6)	0 (0)	0.2
Month 1 response, CR/sCR, *n* (%)	10 (17)	10 (21)	0 (0)	0.07
Best response- CR/sCR, *n* (%)	27 (45)	27 (57)	0 (0)	**<0.001**
FLC < normal, *n* (%)	43 (72)	40 (85)	3 (23)	**<0.001**

*ISS* International scoring system, *ECOG* Eastern Cooperative Oncology Group, *ICANS* Immune effector associated neurotoxicity syndrome, *CR/sCR* Complete response/Stringent complete response, *FLC* Free light chain

*p* values that are statistically significant are shown in bold.

### Serial BM assessment

At month 1, 78% (47/60) of the patients had BM that were MRDneg. Baseline demographics were comparable between BM-MRDneg and BM-MRDpos groups except for the use of bridging therapy (Table 1). IMWG response at month 1 was CR/sCR in 17% (10/60), VGPR in 20% (12/60), PR in 30% (18/60), SD in 23% (14/60), PD in 8% (5/60) and 2% (1/60) patients were not evaluable. The CR/sCR rate at month 1 in the BM-MRDneg group was 21% (10/47) and in the BM-MRDpos group was 0%.

**Fig. 1:**
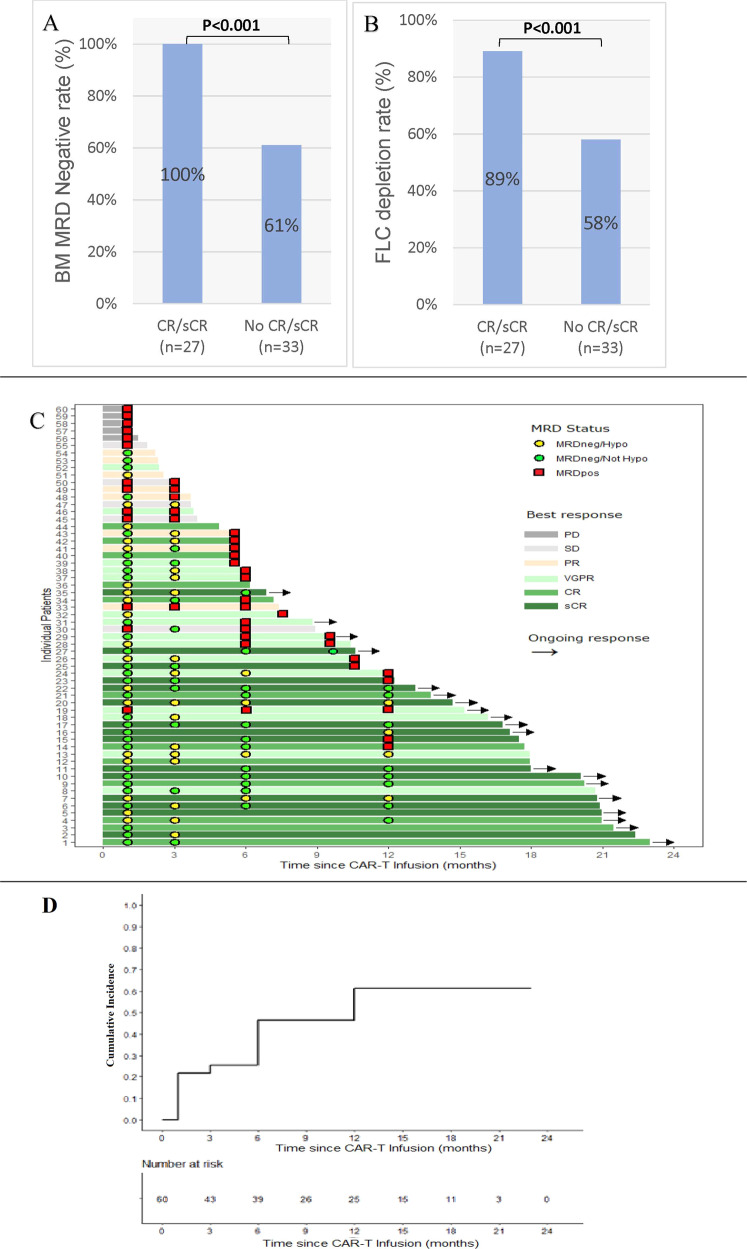
Bone marrow MRD status and associated clinical outcome for patients with myeloma who received CAR-T. Bar graph comparing rates of BM MRDneg rate (**A**) and FLC clearance (**B**) in patients who achieved CR/sCR (as per IMWG) vs those who didn’t. **C** Swimmer’s plot showing progression free survival (PFS) and best response, as per IMWG, among individual CAR-T patients. (Square = MRD positive, Circle = MRD negative), **D** Cumulative incidence curve for BM MRDpos after CAR-T infusion.

Overall, 72% (43/60) patients had FLC < normal (NL) at month 1; with 85% (40/47) patients in the BM-MRDneg group had FLC < NL, and 23% (3/13) in the BM-MRDposgroup. Compared to all other responses, patients who achieved CR/sCR as their best response had higher rates of month 1 BM-MRDneg (100% vs. 61%, *p* < 0.001, Fig. 1A) and FLC < NL (89% vs. 58%, *p* < 0.001, Fig. 1B). Patients who were BM-MRDneg at month 1, 18/47 (38%) patients had hypocellular BM. Cellularity could not be evaluated for 3/47 (6%) patients due to suboptimal BM sample.

BM samples at month 3 (*n* = 35), 6 (*n* = 28) and 12 (*n* = 22) showed BM-MRDneg rates of 74% (26/35), 54% (15/28), and 59% (13/22), respectively. The rate of BM MRDneg/hypocellularity was 62% (16/26), 27%% (4/15), and 31% (4/13), respectively. Among patients who had serial BM evaluation, 82% (47/60) had serial samples. The rate of sustained BM-MRDneg status at month 6 was 34% (16/47) and at month 12 was 28% (13/47, Fig. 1C). All patients except one, who was BM-MRD positive (MRDpos) at month 1 continued to be MRDpos(Fig. 1D). This patient had patchy areas of clonal plasma cells visible on BM biopsy, even though BM aspirate was negative for MRD. By month 3, this patient was BM MRDneg on both pathology and flow. The rate of conversion from BM-MRDpos to BM-MRDneg was 5% (1/20). At month 1, 18/47 (38%) patients were BM-MRDneg/hypocellular and 78% (14/18) of these patients had serial samples. The rate of sustained BM hypocellularity≥12 was 50% (7/14). Recovery to normal cellularity was observed in 50% (7/14) patients and the median time to recovery was 12 (range, 3 –Not reached) months (Fig. 1C). Patients who had continued BM MRDneg achieved a deeper response.

### BM MRD status and prognosis with clinical outcome

**Fig. 2:**
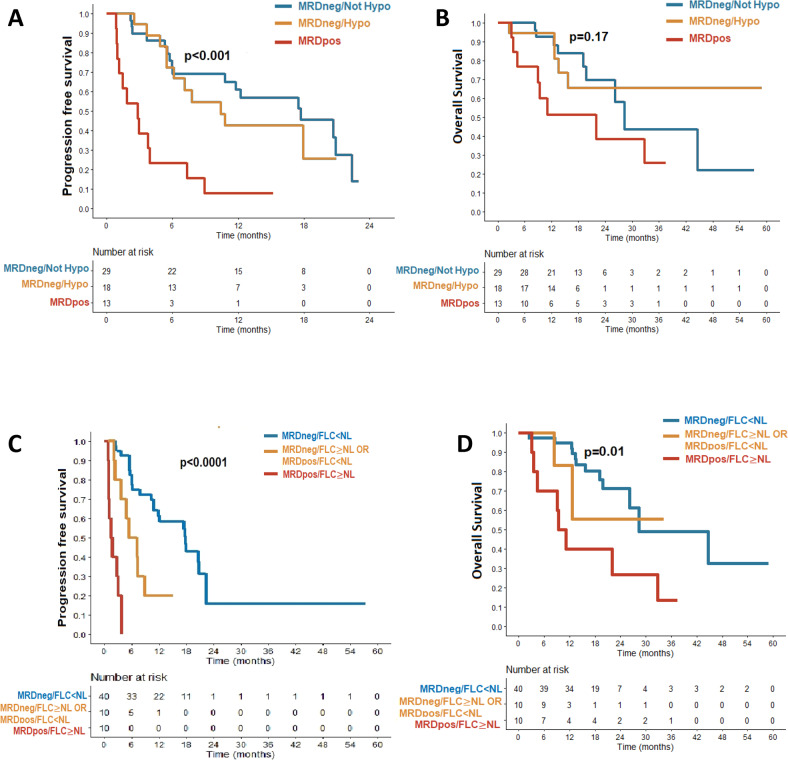
Bone marrow MRD status and correlation with clinical outcome for patients with myeloma who received CAR-T. **A** Kaplan-Meier curve for progression free survival (PFS) between patients with month 1 BM MRDpos and MRDneg stratified by BM cellularity (top left). **B** Kaplan-Meier curve for overall survival (OS) between patients with month 1 BM MRDpos and MRDneg stratified by BM cellularity (top right (**C**) Kaplan-Meier curve) for PFS among patients with month 1 BM MRDneg/FLC < NL, MRDneg/FLC > = NL or MRDpos/FLC < NL and MRDpos/FLC > = NL(bottom right). **D** Kaplan-Meier curve for OS among patients with month 1 BM MRDneg/FLC < NL, MRDneg/FLC > = NL or MRDpos/FLC < NL and MRDpos/FLC > = NL (bottom left).

Compared to BM-MRDpos, patients who had BM-MRDneg at months1 had longer PFS [17.5 months (95% CI, 10.4-NR), *p* < 0.0001 vs. 2.9 months (95% CI, 1.2-NR), Fig. 2A]. PFS was not statistically significantly different between patients who had BM-MRDneg and were either hypocellular or not. [17.7 months vs 10.4, *p* = 0.46]. OS did not reach statistical significance [BM-MRDneg/Not Hypocellular: Not reached vs BM-MRDneg/Hypocellular:28.3 months vs BM-MRDpos:21.9 months, *p* = 0.17, Fig. 2B]. BM-MRDneg patients with FLC < NL at months1 had better median PFS compared to those who did not. (BM-MRDpos/FLC ≥ NL: 1.7 months (0.9-NR); BM-MRDneg/FLC ≥ NL or BM-MRDpos/FLC < NL: 6.3 months (3.7-NR); BM-MRDneg/FLC < NL:17.9 months (11.8-NR), *p* < 0.001, Fig. 2C, Supplementary Fig. 1a). Patients with BM MRDneg and FLC < NL had better OS as compared to all others (BM-MRDpos/FLC > = NL:10.2 months; BM-MRDneg/FLC ≥ NL or BM-MRDpos/FLC < NL:NR months vs BM-MRDneg/FLC < NL:28.3 months, *p* < 0.05, Fig. 2D, Supplementary Fig. 1b).Sustained BM MRDneg ≥12 status with serial biopsy further reinforced the prognostic value. The median PFS for patients who maintained BM MRDneg status at month 6 was 20.7 months (95% CI: 17.9 – NR months); and for patients who maintain BM MRDneg status at month 12 was 20.9 months (95% CI 20.9 – NR months).

## Discussion

BM MRDneg status has been demonstrated to be prognostic for prolonged PFS and OS in drug-based myeloma therapy [2, 9, 10]. In patients receiving CAR-T, we observed BM was hypocellular in one-third of the patients in the first-year post-CAR-T infusion. Regardless of BM cellularity status, BM MRDneg at month 1 and FLC below normal correlated with CR/sCR response and prolonged PFS. BM assessment of MRD and serum FLC levels can be used as a surrogate for the prediction of response and PFS post-CAR-T. This is the first study to report a correlation of BM cellularity and BM MRDneg status with treatment outcomes in patients receiving CAR-T.

Patients who had serum FLC < NL and were BM-MRDneg at month 1, regardless of IMWG response criteria at the time, had the longest survival in this cohort (median 17.9 months); and those who had serum FLC at or above normal and were BM-MRDpositive at month 1 had the shortest survival. Those who were either FLC at or above normal OR BM MRDpos had an intermediate duration of PFS. Larger studies are needed to fully delineate the prognosis for either finding within this category.

While we also observed that sustained serial BM MRDneg ≥12 is associated with durable response, an early MRD assessment at month 1 can potentially predict for risk of relapse and be used for risk-adapted monitoring. Patients with a rapid tumor clearance and early BM MRD negativity but not CR by IMWG criteria may later evolve to complete response. This is likely the reason that clearance of serum FLC at month 1, which has a much shorter half-life than monoclonal proteins, is also associated with deeper IMWG response and prolonged PFS.

Patients who were MRD positive at month 1 have a low likelihood of achieving MRDneg. Thus, early MRD positivity likely predicts impending clinical relapse. Because the majority of these patients who are MRDpos at month 1 do not convert to MRDneg and have significantly shorter PFS, clinical trials should be designed to incorporate these high-risk patients to understand the biology of resistant clones and inform strategy for early intervention.

Sustained MRDneg status at months 6 and 12 have been persistently found to be prognostic for prolonged PFS in other myeloma therapies [11]. Our serial BM MRD data in CAR-T patients corroborates these findings. This support continued adherence of IMWG recommendation for the testing schedule of BM MRD for patients who receive CAR-T. However additional study for the role of BM MRD study at the early timepoint of month 1 along with serum FLC could be useful given the kinetics of action and the management logistics of this therapy.

The first limitation of our study is the small sample size. Larger, future prospective studies can help validate our findings and help determine the optimal timing for MRD assessment post-CAR-T. BM assessment of MRD may sometimes be limited by the quality of BM aspirate (patchy or dilute), MRD detection methods used, and the risk of false-negative results due to extramedullary disease. The second limitation is that our study uses only one test i.e., flow cytometry for MRD assessment. NGS may enable more sensitive detection of MRDneg threshold. Non-invasive MRD testing like PET-CT, circulating tumor DNA, and serum BCMA levels can also help with surveillance monitoring. The incorporation of these tools to help assess global MRD status are ongoing. Our data support the continued evaluation of BM and also serum FLC levels early post CAR-T infusion as a prognostic tool. Given the higher occurrence of extramedullary disease in highly pre-treated patient population, a blood-based MRD testing such as NGS, circulating tumor DNA, MALDI-TOF, PET-CT may have utility in the global assessment of risk.

## Supplementary information

Supplementary

## Data Availability

The data that supports this study is available on request from the corresponding author upon reasonable request.
