# Supplementary material for: Prognostic value of early bone marrow MRD status in CAR-T therapy for myeloma
Source: Blood Cancer J. 2023 Apr 5;13(1):47. doi: 10.1038/s41408-023-00820-y (PMC10076306; doi:10.1038/s41408-023-00820-y)

Supplementary Table

Figure 1: Kaplan meier curve for (A) progression free survival and (B) overall survival comparing BM-MRD/FLC neg and pos patients


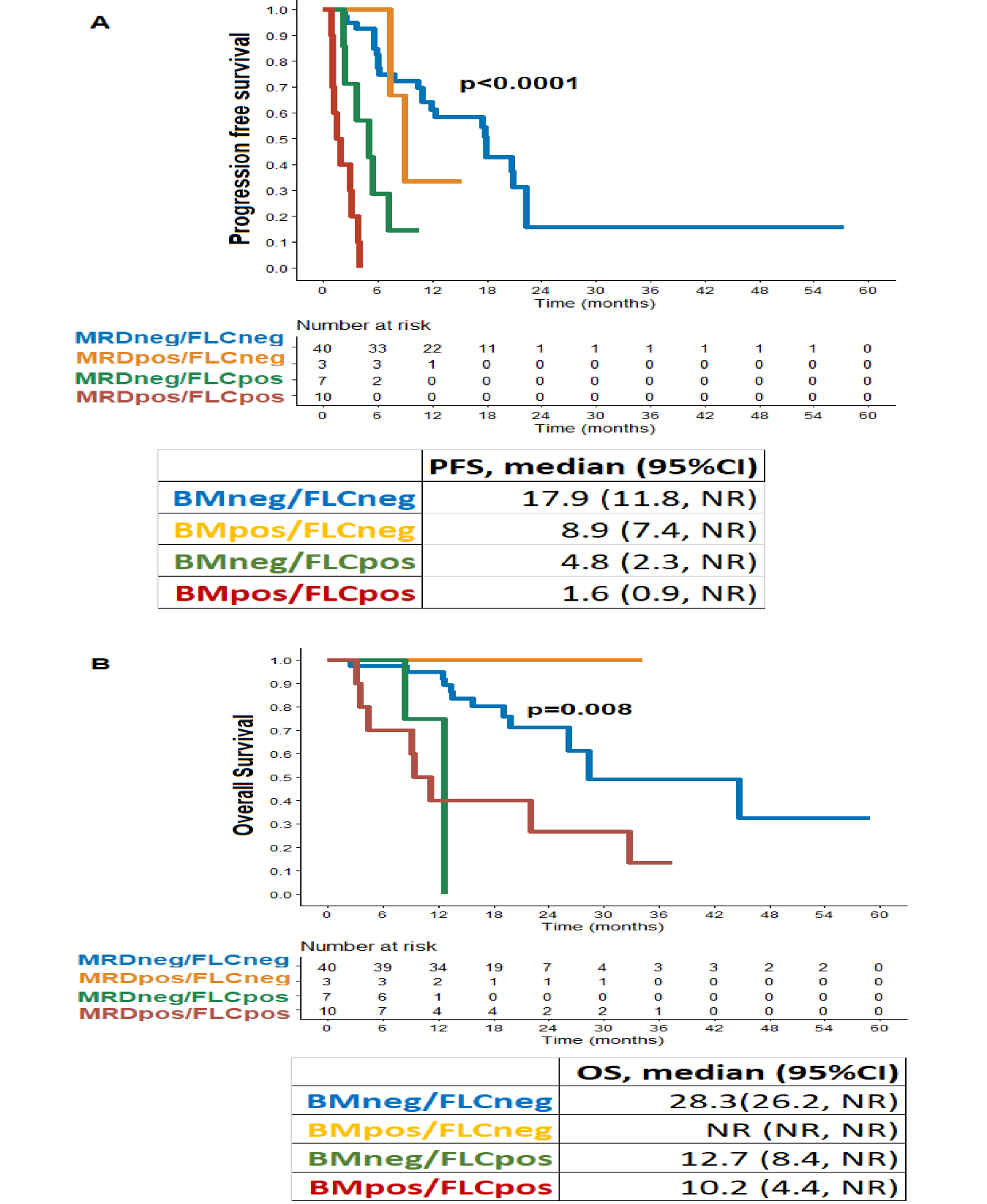

Supplement: Supplementary file 1 — Supplementary [file 41408_2023_820_MOESM1_ESM.docx]
